# Supplementary material for: Multi-Evaluating Strategy for Siji-kangbingdu Mixture: Chemical Profiling, Fingerprint Characterization, and Quantitative Analysis
Source: Molecules. 2019 Sep 30;24(19):3545. doi: 10.3390/molecules24193545 (PMC6804210; doi:10.3390/molecules24193545)
Supplement: Supplementary file 1 [file molecules-24-03545-s001.pdf]

Multi-evaluating strategy for Siji-kangbingdu Mixture: chemical profiling, fingerprint characterization and quantitative analysis

Zhuoru Yao [1#], Jingao Yu [1*#], Zhishu Tang [1*], Hongbo Liu [1*], Kaihua Ruan [1], Zhongxing Song [1], Yanru Liu [1], Kun Yan [2], Yan Liu [2], Yuping Tang [2] and Huqiang Ma [3]

[1] Shaanxi Collaborative Innovation Center of Chinese Medicine Resources Industrialization/ State Key Laboratory of Research & Development of Characteristic Qin Medicine Resources (Cultivation)/ Shaanxi Innovative Drug Research Center, Shaanxi University of Chinese Medicine, Xianyang, 712000, China; yaozhuoru0708@gmail.com (Z.Y.); rkh15709100891@163.com (K.R.); szx74816@sina.com (Z.S.); 1501011@sntcm.edu.cn (Y.L.).

[2] College of pharmacy, Shaanxi University of Chinese Medicine, Xixian New Area, 712046, China; 18791029030.@139.com (K.Y.); ly712537@163.com (Y.L.); yupingtang9@126.com (Y.T.).

[3] Shaanxi Haitian pharmaceutical co., LTD, Xixian New Area, 712046, China; mahuqiang_2016@163.com (H.M.)

* Correspondence: jingao_yu@sina.cn (J.Y.); or tzs6565@163.com (Z.T.), or 1501010@sntcm.edu.cn (H.L.).

# These authors contributed equally to this work.

Supplementary information

Table S1. Chemical compounds identified by UPLC-TripleTOF-MS technology coupled with searching algorisms against TCM reference material library.

NO.	RT (min)	Positive ion mode				Negative ion mode				Formula	Identified compound	Other possible compounds	Compound type
		Mass (Da)	Mass error (ppm)	Library Score	Area	Mass (Da)	Mass error (ppm)	Library Score	Area				
1	0.49	164.09173	-4.4	--	490	--	--	--	--	C6H13NO4	1-Deoxynojirimycin	--	other
2	0.65	--	--	--	--	191.0197	1.2	89.3	1481	C6H8O7	Citric acid	--	Organic acid
3	0.68	--	--	--	--	827.2674	0.2	--	93	C30H52O26	Maltopentaose	--	Oligosaccharide
4	0.71	--	--	--	--	243.0623	1.5	--	57	C9H12N2O6	Uridine	--	Nucleoside
5	0.72	268.10402	0.7	99.6	24264	--	--	--	--	C10H13N5O4	Adenosine	--	Nucleoside
6	0.75	150.09134	-3.1	37.6	1829	--	--	--	--	C9H11NO	4-Dimethylaminobenzaldehyde	--	other
7	0.79	284.09895	0.9	100	10979	282.0844	0.9	99.7	66	C10H13N5O5	guanosine	--	Nucleoside
8	1.34	166.08626	-1.3	80	30107	164.0717	1	97.9	161	C9H11NO2	Phenylalanine	--	Amino acid
9	1.37	127.03897	-4.4	--	3773	--	--	--	--	C6H6O3	5-Hydroxymethylfurfural	--	other
10	2.52	195.06518	-1.6	--	833	--	--	--	--	C10H10O4	Ferulic Acid	Isoferulic acid	Phenolic acid
11	2.53	359.13365	-0.5	55.1	15690	--	--	--	--	C16H22O9	sweroside	--	other
12	2.58	--	--	--	--	375.1297	0.8	56.9	6263	C16H24O10	8-epiloganic acid	Vitamin B2, Loganic acid	Monoterpene
13	3.08	--	--	--	--	137.0244	0.9	84.3	877	C7H6O3	Protocatechuic aldehyde	4-Hydroxybenzoic acid, salicylic acid, Sesamol	other
14	3.15	--	--	--	--	203.0826	3	94.7	172	C11H12N2O2	L-Tryptophan	--	Amino acid
15	3.58	318.15474	0.3	69.7	57571	345.1191	0.8	93.4	1694	C14H20O7.NH3	Salidroside +NH3	salidroide+NH3, Aucubin	Phenylpropanoid glycoside
16	5.88	179.03389	0.4		1461	177.0193	1.5	48.5	236	C9H6O4	Daphnetin	--	
17	6.34	355.10237	-0.4	100	37856	353.0878	0.9	99.9	2454	C16H18O9	Chlorogenic acid	--	Phenolic acid

18	10.82	183.06518	-1	62.9	1443	--	--	--	--	C9H10O4	Syringaldehyde	3,4-Dimethoxybenzoic acid, Homovanillic acid	other
19	11.45	--	--	--	--	403.1246	0.8	65.6	177	C17H24O11	Gardenoside	sweroside +HCOOH	Monoterpene glycoside
20	11.94	623.19703	-0.2	32.2	463	--	--	--	--	C29H34O15	Pectolinarin	--	Flavonoid glycoside
21	12.22	--	--	--	--	593.1512	4	35.4	452	C27H30O15	Glucosylvitexin	Aempferol-3-O-rutinoside, Oroxin B	Flavonoid glycoside
22	12.22	--	--	--	--	389.1453	0.5	48.9	287	C17H26O10	Loganin	--	Monoterpene glycoside
23	14.2	149.0961	-0.7	48.6	1590	--	--	--	--	C10H12O	Anise oil	--	other
24	14.32	700.28114	-1.3	99.2	5145	--	--	--	--	C32H42O16.NH3	Pinoresinol Diglucoside +NH3	--	Diterpene glycoside
25	15.4	273.07576	-0.6	96.2	1835	--	--	--	--	C15H12O5	Naringenin	--	Flavonoid
26	15.83	565.15516	-0.9	98.3	17364	563.1406	4.6	99.4	427	C26H28O14	Schaftoside	Isoschaftoside	Flavonoid glycoside
27	16.62	419.13365	-0.2	99.7	15576	417.1191	2.2	94.6	2916	C21H22O9	Liquiritin	--	Flavonoid glycoside
28	21.67	611.16066	0	99.4	61425	--	--	--	--	C27H30O16	Rutin	--	Flavonoid glycoside
29	21.71	465.10277	-0.8	100	19872	463.0882	4.4	99.7	314	C21H20O12	Hyperin	myricitrin, Isoquercitrin	Flavonoid glycoside
30	22.03	463.08711	-0.6	100	31099	461.0726	3.3	100	641	C21H18O12	Luteolin-7-O-β-D-glucuronide	Scutellarin	Flavonoid glycoside
31	22.51	449.10783	-0.3	100	16039	--	--	--	--	C21H20O11	Astragalin	Luteoloside, Quercitrin, Quercetin 7-rhamnoside, Cyanidin-3-O-glucoside, Rhodionin, Orientin	Flavonoid glycoside
32	23.84	--	--	--	--	577.1563	3.3	47.4	231	C27H30O14	Rhoifolin	Kaempferitrin, Vitexin-2-O-rhamnoside	Flavonoid glycoside
33	25.18	--	--	--	--	477.1403	4.5	100	2818	C23H26O11	Calceorioside B	--	Phenylpropanoid
34	25.61	642.23927	-1	84.2	312924	623.1982	4.8	95.3	47276	C29H36O15.NH3	Forsythoside A +NH3	--	Phenylpropanoid
35	26.2	774.28151	-0.4	98.8	12373	--	--	--	--	C34H44O19.NH3	Forsythoside B +NH3	--	Phenylpropanoid

36	26.52	303.04994	0.7	96.3	21822					C15H10O7	Quercetin dihydrate	Morin hydrate	Flavonoid
37	26.54	287.05501	-0.5	98.3	3461	--	--	--	--	C15H10O6	Luteolin	--	Flavonoid
38	26.61	517.13405	-0.7	98.8	26168	515.1195	1	100	6535	C25H24O12	Isochlorogenic acid A	--	Phenolic acid
39	27.56	433.11293	-1	99.7	32368	431.0984	3.4	99	836	C21H20O10	Apigenin-7-glucoside	Emodin-8-glucoside, Isovitexin, Genistin, Sophoricoside, Vitexin, Afzelin	Flavonoid glycoside
40	28.14	447.09218	-1	99.2	9933	445.0776	4.8	99.7	229	C21H18O11	Apigenin 7-O-beta-D-glucuronide	Baicalin	Flavonoid glycoside
41	29.12					441.1766	3.4	96.2	417	C20H28O8.HCOOH	Lobetyolin +HCOOH	--	other
42	30.78	611.19703	-2	95.5	21549					C28H34O15	hesperidin	Neohesperidin, Hesperetin	Flavonoid glycoside
43	32.55					359.0772	4.4	98	4599	C18H16O8	Rosmarinic acid	--	Phenolic acid
44	32.67	431.13365	0	99.3	66834	--	--	--	--	C22H22O9	Ononin	--	Flavonoid glycoside
45	33.11	255.06518	-0.9	85.6	3536					C15H10O4	Chrysin	Daidzein, Chrysophanol	Flavonoid
46	33.23	463.12349	-0.3	96.9	13513	--	--	--	--	C22H22O11	Pratensein-7-O-glucoside	Daidzin +HCOOH, Tectoridin	Flavonoid glycoside
47	34.35	289.07066	-0.5	89.8	1038					C15H12O6	Eriodictyol	--	Flavonoid
48	35.15	287.09141	-0.4	46.4	6883	--	--	--	--	C16H14O5	Kaempferol	--	Flavonoid
49	35.4	257.08082	0.8	93.5	68154					C15H12O4	Liquiritigenin	--	Flavonoid
50	39.68	285.07576	-0.8	85.7	9643	--	--	--	--	C16H12O5	Calycosin	Physcion, Wogonin, Genkwanin, Acacetin	Flavonoid
51	40.29	552.24391	-0.7	41	540118	--	--	--	--	C27H34O11.NH3	arctiin +NH3	--	Phenylpropanoid
52	40.55	--	--	--	--	579.2085	0.5	100	16863	C27H34O11.HCOOH	Phillyrin\Forsythin +HCOOH	--	Phenylpropanoid
53	41.86	593.18648	-1	99.5	32005	--	--	--	--	C28H32O14	linarin	--	Flavonoid glycoside
54	42.07	447.12858	-0.5	99.4	29095	--	--	--	--	C22H22O10	Glycitin	calycosin-7-o-glucoside	Flavonoid glycoside

55	42.41	331.08122	-0.1	39.1	4057	--	--	--	--	C17H14O7	Aurantio-obtusin	--	Anthraquinone
56	42.49	271.0601	0.4	90.5	395	--	--	--	--	C15H10O5	Emodin	--	Anthraquinone
57	42.84	247.0601	-0.7	94	351	--	--	--	--	C13H10O5	Isopimpinellin	--	Coumarin
58	43.28	153.12739	-3	65.3	3593	--	--	--	--	C10H16O	Pulegone	--	Monoterpene
59	43.94	345.09688	0.5	90	1097	--	--	--	--	C18H16O7	Eupatilin	Usnic acid, Lysionotin	Flavonoid glycoside
60	44.26	269.08082	0.4	91	17010	--	--	--	--	C16H12O4	Formononetin	--	Flavonoid
61	44.41	373.16456	-0.7	34.1	5470	--	--	--	--	C21H24O6	Arctigenin	--	Phenylpropanoid
62	46.52	315.08632	-0.4	75.5	2788	--	--	--	--	C17H14O6	Pectolinarigenin	--	Flavonoid
63	48.08	375.10744	0	87.4	18921	--	--	--	--	C19H18O8	Chrysosplenetin B	vitexicarpin	Flavonoid
64	48.46	403.13875	-0.3	95	33677	--	--	--	--	C21H22O8	Nobiletin	--	Flavonoid
65	48.61	823.41109	-0.7	99.8	100668	--	--	--	--	C42H62O16	Glycyrrhizic acid	--	Triterpene saponins
66	51.43	457.36763	-0.9	54.7	1123	--	--	--	--	C30H48O3	Betulinic acid	Ursolic Acid, Oleanolic acid	Triterpene
67	51.47	322.10737	0.8	85.9	6004	--	--	--	--	C19H15NO4	Berberrubine	--	other
68	51.64	455.35197	-2.7	57.5	13517	--	--	--	--	C30H46O3	Betulonicacid	--	Triterpene
69	51.75	373.12819	0.7	93.2	18745	--	--	--	--	C20H20O7	Tangeretin	--	Flavonoid
70	52.21	245.11722	-1	91.1	2652	--	--	--	--	C15H16O3	Osthole	--	Coumarin

Note 1: The“--” signal means not detected or beyond the confidential level. The 7 determined compounds marked in red were verified with their reference materials. In this table, contents in the “Formula” and “Identified compound” column were both inherited from the compound library. Take the compound of NO.15 as an example, the name “Salidroside +NH3” actually means the “Salidroside + [NH4]+” adducts; and the formula “C14H20O7.NH3” actually means “C14H20O7.NH4”. In TCM compounds, some glycosides have intensive tendencies to form the ammonium adducts rather than proton adducts. Although we did not add ammonium to the eluent, the ammonium ion may be brought in by the solvent system.

Table S2. The fingerprint technical parameters of 49 common peaks.

No.	Retention time /min	Relative retention time	Peak Area	Relative Peak Area	Peak Area RSD (%)	Peak identification
1	1.733	0.070	499464.9±63345.75	0.218±0.026	12.68	
2	2.087	0.084	690488.7±55953.35	0.303±0.028	8.10	
3	2.444	0.098	509525.4±85193.04	0.223±0.034	16.72	
4	2.963	0.119	1569795.5±221970.4	0.687±0.096	14.14	
5	3.240	0.130	369957.3±30202.25	0.162±0.011	8.16	
6	3.601	0.145	359016.5±20399.48	0.157±0.009	5.68	
7	3.999	0.161	36282.4±9448.53	0.016±0.004	26.04	
8	4.806	0.193	83637.8±21243.45	0.036±0.009	25.40	
9	5.363	0.216	83792.2±25526.88	0.037±0.011	30.46	
10	5.803	0.234	128320.4±21772.68	0.056±0.011	16.97	
11	6.834	0.275	909965.1±95027.7	0.397±0.031	10.44	
12	7.490	0.302	192864.1±10350.03	0.084±0.005	5.37	Chlorogenic acid
13	8.340	0.336	41680.6±7039.46	0.018±0.003	16.89	
14	9.473	0.381	629761.7±57728.37	0.276±0.024	9.17	
15	10.065	0.405	275298.9±39392.39	0.120±0.016	14.31	
16	10.413	0.419	398811.5±109365.4	0.174±0.044	27.42	
17	10.962	0.441	477615.3±86745.12	0.208±0.034	18.16	
18	11.368	0.458	123989.6±39434.37	0.054±0.016	31.80	
19	11.557	0.465	108652.7±22113.71	0.047±0.009	20.35	
20	11.940	0.481	4319730.5±341679	1.892±0.169	7.91	
21	13.233	0.533	66713.6±10347.49	0.029±0.005	15.51	
22	16.136	0.650	220119.1±52432.55	0.096±0.021	23.82	Liquiritin

23	16.889	0.680	77168.9±5583.51	0.034±0.002	7.24	
24	18.403	0.741	93078.2±36909.59	0.041±0.015	39.65	
25	18.889	0.760	2125304.6±119502.9	0.929±0.020	5.62	
26	19.766	0.796	114610.9±17207.57	0.050±0.008	15.01	
27	21.091	0.849	502702.9±35440.68	0.220±0.008	7.05	Rutin
28	22.149	0.892	72667.6±20247.45	0.032±0.009	27.86	
29	22.673	0.913	440705.4±53027.2	0.193±0.024	12.03	
30	23.031	0.927	73845.8±49504.29	0.032±0.021	67.04	
31	23.816	0.959	3069796.5±232043.4	1.342±0.069	7.56	
32	24.35	0.980	183898.1±66353.52	0.081±0.028	36.08	
33	24.839	1.000	2287000.1±102839.4	1.000±0.000	4.50	Forsythoside A
34	25.992	1.046	369022.1±127531.2	0.160±0.050	34.56	Isochlorogenic acid A
35	27.059	1.089	9754567.9±185277.2	4.272±0.175	1.90	
36	31.311	1.261	101198.5±69506.52	0.044±0.029	68.68	
37	31.792	1.280	52480.9±13009.09	0.023±0.005	24.79	
38	32.376	1.303	234826.9±48326.21	0.102±0.018	20.58	
39	33.558	1.351	263980.6±22472.54	0.115±0.008	8.51	
40	38.960	1.569	179087.2±13675.97	0.078±0.006	7.64	Forsythin
41	41.841	1.684	6215596.5±376617.6	2.726±0.250	6.06	
42	43.308	1.744	236365.6±33869.87	0.103±0.014	14.33	
43	44.963	1.810	108794.1±31980.54	0.048±0.013	29.4	
44	46.509	1.872	78226.1±30500.72	0.034±0.013	38.99	
45	47.082	1.895	27981.6±7973.32	0.012±0.003	28.49	
46	47.84	1.926	763361.5±251866.8	0.333±0.104	32.99	Glycyrrhizic acid
47	51.538	2.075	271272.2±68569.16	0.119±0.028	25.28	
48	52.656	2.120	162106.8±19050.67	0.071±0.007	11.75	

49	53.267	2.144	54907.8±3559.47	0.024±0.001	6.48

Note: Forsythoside A is selected as internal referring substance. Peak area data are showed as Mean ± SD (n=15)

Table S3. Peak areas of seven determined characteristic peaks.

No.	Peak Area of Seven Characteristic Peaks						
	**Chlorogenic acid**	**Liquiritin**	**Rutin**	**Forsythoside A**	**Isochlorogenic acid A**	**Forsythin**	**Glycyrrhizic acid**
S1	196073	185853	456786	2773590	2164644	179173	598046
S2	204644	189657	461382	2798250	2182897	178768	596304
S3	190834	171477	459600	2673302	2111794	180417	560860
S4	185382	167141	456291	2785827	2138218	186709	561084
S5	205851	185282	546245	3077614	2357087	181314	600715
S6	202537	182657	547892	3415166	2351951	185224	602931
S7	192710	185056	528560	3408203	2348199	196443	599253
S8	204679	200351	540035	3381807	2361915	199336	598264
S9	197424	185367	533278	3184572	2404242	192863	604548
S10	200825	196679	542989	3160885	2372919	181769	604517
S11	191209	297988	501556	3081322	2378652	174575	1147331
S12	181364	296062	503039	3045850	2377288	175365	1143579
S13	189877	301076	606171	3085141	2330675	159879	1139668
S14	176542	277747	478155	3076948	2221574	168405	1046988
S15	173009	279392	478562	3098472	2202945	146066	1046333
C.V. (%)	5.366	23.820	8.719	7.559	4.497	7.637	32.994

Note: C.V. means coefficient of variation.

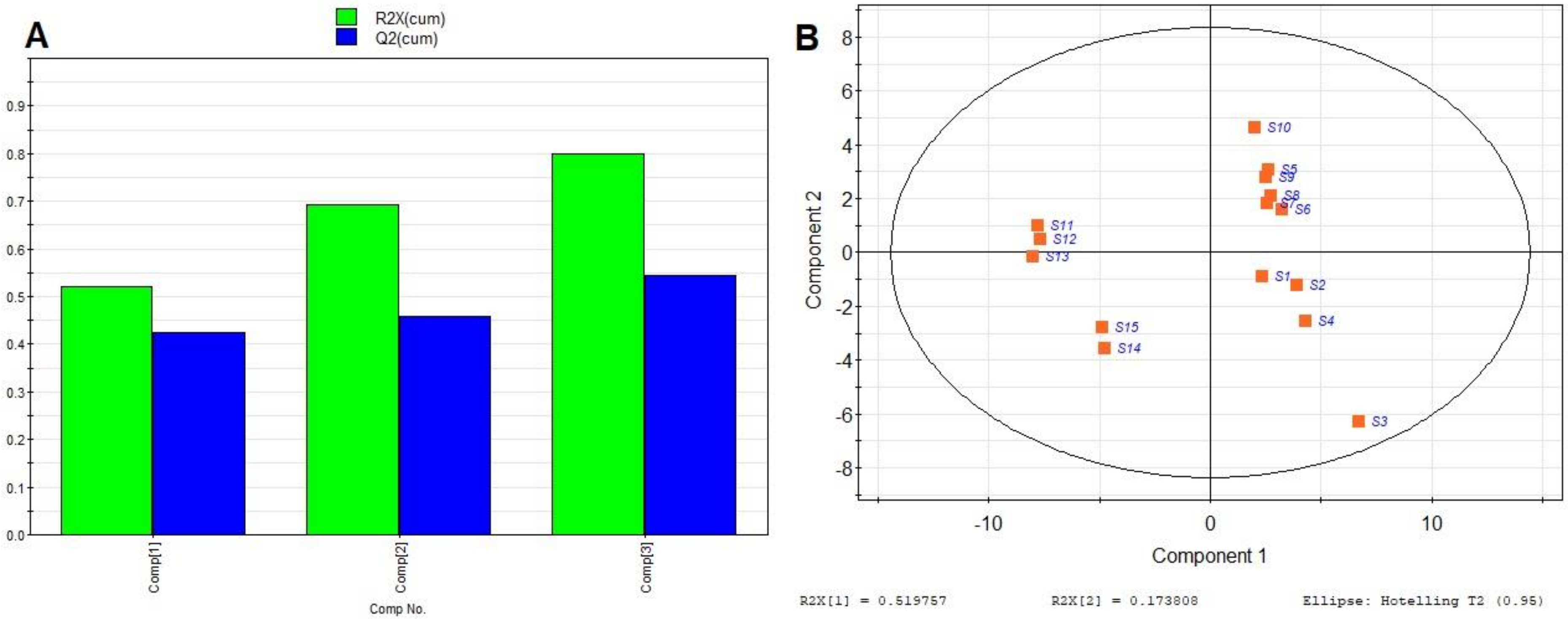

Figure S1. The PCA analysis of fifteen batches of samples S1-S15 based on 49 chromatographic peaks. (A) Model overview plot. Cumulated by 3 components, the R2X value is near 0.8, and the Q2 value is near 0.55. (B) Scatter plot of fifteen samples.
